# Community knowledge, perceptions and water contact practices associated with transmission of urinary schistosomiasis in an endemic region: a qualitative cross-sectional study

**DOI:** 10.1186/s12889-019-7041-5

**Published:** 2019-06-07

**Authors:** Teckla Angelo, Safari M. Kinung’hi, Jorum Buza, Joseph R. Mwanga, Henry Curtis Kariuki, Shona Wilson

**Affiliations:** 1 Department of Global Health and Bio-Medical Sciences, School of Life Sciences and Bioengineering, Nelson Mandela African Institution of Science and Technology (NM-AIST), P. O. Box 447, Arusha, Tanzania; 2 National Institute for Medical Research (NIMR), Mwanza Centre, P. O. Box 1462, Mwanza, Tanzania; 3 Kenya Methodist University, School of Medicine and Health Sciences, P. O. Box 267, Meru, Kenya; 4 Department of Pathology, University of Cambridge, Tennis Court Road, Cambridge, CB2 1QP UK

**Keywords:** Perceptions, Urinary schistosomiasis, Water contact, Practices

## Abstract

**Background:**

In an effort to complement the current chemotherapy based schistosomiasis control interventions in Shinyanga district, community knowledge, perceptions and water contact practices were qualitatively assessed using focus group discussions and semi structured interviews involving 271 participants in one *S. haematobium* prevalent community of Ikingwamanoti village, Shinyanga district, Northwestern, Tanzania.

**Methods:**

In October, 2016 we conducted 29 parent semi structured interviews and 16 focus group discussions with a total of 168 parent informants. Adult participants were conveniently selected from three sub-villages of Butini, Miyu, and Bomani of Ikingwamanoti village, Shinyanga district. In March, 2017, a total of 103 children informants participated in 10 focus group discussions and 20 semi structured interviews, administered to children from standard four, five, six and seven attending Ikingwamanoti Primary School. Note taking and digital recorders were used to collect narrative data for thematic analysis of emergent themes.

**Results:**

Among participants, 75% parents and 50% children considered urinary schistosomiasis as a low priority health problem. Of the informants, 70% children and 48.3% parents had misconceptions about the cause, modes of transmission and control of schistosomiasis demonstrating gaps in their biomedical knowledge of the disease. Assessment of treatment seeking behavior for urinary schistosomiasis revealed a combination of traditional and modern health care sectors. However, modern medicines were considered effective in the treatment of urinary schistosomiasis. Lack of alternative sources of water for domestic and recreational activities and unhygienic water use habits exposed community members to high risk of acquiring urinary schistosomiasis.

**Conclusion:**

Use of *Schistosoma haematobium* contaminated water sources for daily domestic and recreational use facilitated contraction of urinary schistosomiasis among community members in Shinyanga district. People’s perceptions of urinary schistosomiasis as a less priority health problem promoted persistence of the disease. Future efforts to control urinary schistosomiasis should take into account integrated approaches combining water, sanitation and hygiene, health education, alternative sources of clean and safe water to facilitate behavior change.

**Electronic supplementary material:**

The online version of this article (10.1186/s12889-019-7041-5) contains supplementary material, which is available to authorized users.

## Background

Schistosomiasis is an infectious disease affecting more than 230 million people worldwide [1]. An estimated 95% of cases occur in Africa [2–5]. It is caused by six blood fluke species of the genus *Schistosoma* [6]. Transmission of the parasite depends on unhygienic conditions, in which human excreta directly contaminate freshwater bodies that are habitats for specific snail intermediate hosts [7]. The disease is mainly transmitted in rural settings where there is insufficient sanitation, poor health awareness and poverty [8]. Measures to control and eventually eliminate the disease through preventive chemotherapy have been in place for several years in most African endemic countries using praziquantel as the drug of choice. However the strategy has proved to have limitations where there is persistent re-infection due to praziquantel’s inability to kill immature worms and prevent re-infection [1, 9]. In Tanzania, schistosomiasis remains a serious public health problem. Despite of ongoing control interventions, high prevalence of the disease still persists [10]**.** The 65th World Health Assembly recommendation states that endemic countries must strengthen their local control programmes and enable provision of at least a single annual round of praziquantel treatment to school age children [11]. However, the chemotherapy approach alone in most endemic areas like Tanzania has not yielded a sustainable impact as people continue to enter water containing infective cercariae, re-infection following praziquantel treatment has been common. Therefore additional interventions are necessary to facilitate progress towards control and elimination [12–15]. These include, water based interventions such as water sanitation and hygiene (WASH) and mollusciciding [16, 17]. Further, correct knowledge, attitude and practices of community members in endemic areas have a significant role to play in achieving success of the planned control interventions and achieve sustainable control of the disease [18]. Health education and encouragement of behavioral change, in addition to a chemotherapy approach, are likely to become key elements in future elimination efforts [19–22]. Even though introduction of alternative water supply in resource limited settings is expensive and sometimes lacks priority [16]**,** reduction of contact with infectious water as one of the schistosomiasis control strategies is even more complicated in these settings, requiring a community adapted approach to facilitate control and elimination. To generate effective behavioral change interventions and encourage acceptance of protective behaviors and reduction of risk behaviors, understanding the community knowledge is critical [23]**.** Here we present results of a qualitative study implemented in adults and school children to facilitate the design of community based disease control interventions.

## Methods

### Study area and population

This study was conducted among community members (parents/guardians and school children) living in Ikingwamanoti village, Shinyanga District in Northwestern Tanzania between October 2016 and March 2017. The village has one school, Ikingwamanoti Primary School with 517 enrolled school children. The village lies 02°65,283′ South of the Equator and 32°64,063′ East of the Greenwich Meridian. The village is located along the highway to Tabora region, connecting Mwanza and Dar Es Salaam. The population size of Butini sub village was 783, Bomani sub village 753, and Miyu sub village 563, totaling to 2099 inhabitants for the whole Ikingwamanoti village [22]. The district is predominantly inhabited by *Wasukuma* ethnic group (Bantu-speaking people) who are the natives of the *Sukumaland* located to the west and south of the Lake Victoria. The district receives two phases of rainfall, the short rainy season from November to December and a long rainy season from March through May with rain almost every day. The district is endemic to *S. haematobium* infection with transmission occurring in temporary pools throughout the region [24, 25]. The major income generating activities are largely based on subsistence farming and livestock–keeping. Crops that are cultivated include cassava, millet, yams and paddy which constitutes the food crops whereas cotton is the main cash crop. Crop production and livestock-keeping are undertaken within the framework of individual households. More details of the study area and quantitative analysis of school-children’s *S. haematobium* infection levels and their infection-risk behaviours is described in Angelo et al. [25].

### Study design and sampling procedure

The study was a descriptive qualitative inquiry that was designed to complement quantitative findings of a larger parasitological and malacological study conducted in the same area**.** For adult community participants, a local community leader of each sub village (Butini, Bomani and Miyu) was asked to invite community members to participate in the study by blowing a whistle one day before the event. During the next day, participants who responded the previous day’s call and were willing to participate, gathered in open grounds from which convenient sampling was used to select participants to be recruited into the study. For school children, the research team with the assistance of class teachers selected participants from standard four to seven and included them into the study. Sample size determination was mainly based on salience of ideas and themes for thematic analysis of revealed outcomes rather than reaching saturated recording of all beliefs within the community as described by Weller and others [26] whereby only 10 interviews and further probing are sufficient to capture on average 95% of salient ideas. To capture salient perceptions from individual experiences with urinary schistosomiasis, semi structured interviews were conducted with 29 adult community members and 20 primary school children. Sixteen focus group discussions with adult community members, totaling 168 participants and ten FGDs, with a total of 103 school children were administered to enable generation of community ideas on urinary schistosomiasis.

### Data collection methods

To enable comparison of experiences and creation of new ideas formed from a socio context about urinary schistosomiasis transmission, focus group discussions (FGD) with pre determined questions (Additional file 1) were administered to homogenous groups with similar experiences of urinary schistosomiasis practices. Discussions were conducted in quiet places and comprised of circular seating groups guided by moderators [27, 28]. To investigate individual experiences of school aged children and adult community members a semi structured interviews (SSI) (Additional file 2) with standardized predetermined list of questions were used to solicit information on schistosomiasis knowledge, attitude and practices among community members (adults and school aged children). The rationale of using two different strategies in data collection in this study (focus group discussion and semi structured interviews) was to apply methodological triangulation in order to improve the capture of knowledge, attitudes, perceptions and practices on schistosomiasis within the community and compare the results generated from the two strategies [29]. Semi structured interviews explore more on individual views, perceptions and practices on urinary schistosomiasis, while focus group discussions complement SSI by providing in-depth exploration about community knowledge, attitude, perceptions and practices on urinary schistosomiasis. Either approach is limited in terms of their ability to generalize findings to the whole population mainly because of the small numbers of people participating and the likelihood that the participants will not be a representative sample.

The size of focus group discussion ranged from 8 to 12 participants composed of individuals of the same sex and other aspects emphasized in FGD literature, [27, 30, 31]. Male FGDs were conducted by two trained males one being a moderator and the second being the recorder. In the female focus group discussions, two trained females facilitated the discussion, one being a moderator while the other recorded the information. The interviews and discussions were conducted in Kiswahili, a national language, using a simple topic guide with open ended questions and probes to allow in-depth investigation of the topic. Permission to record the discussions was sought prior to the session. In focus group discussions, participants were assigned unique numbers instead of names to use when contributing their views. Participants were encouraged to speak their views freely and spontaneously. Discussions were held in natural settings outside, under the shade of trees and in quiet places. Data was documented by both note taking and digital recordings.

### Data management and analysis

All textual data were categorized into selected topics, thematic analysis was conducted by classifying all the FGD and SSI responses into specific themes, where cases did not fit the emergent theories, the theories were revised. Digitally recorded information was not typed but used to improve field notes prepared from interviews and focus group discussions in the form of hand written reports. Content analysis was executed by comparing raw data and summaries using the principles of grounded theory [32]. To support the findings, quotes were taken from recorded materials [33]. Investigators assigned more or less the same meaning to the data. Inter-investigator reliability was high as data from FGDs and SSIs validated each other. The study employed explicit, systematic and reproducible methods to increase validation and reliability of the findings [34]. Percentages given are of reported perceptions from the SSIs only, with FGD data used to provide further in-depth exploration. When a perception was reported by 20% or more of the respondents, it was considered salient.

### Ethical considerations

In 2015, the Medical Research Coordination Committee (MRCC) of the National Institute for Medical Research (NIMR), Tanzania received and approved the study (ethics clearance certificate no. NIMR/HQ/R.8a/Vol.IX/2107). Additional approval was made by the University of Cambridge Human Biology Research Ethics Committee (HBREC.2015.28) to conduct this study in Shinyanga district, Tanzania. The study objectives, data collection procedures, potential risks and benefits were explained to participants, local leaders, school administrations before administering interviews and discussions. Adequate time was given to participants to ask questions about the study and it was highlighted that participation was an individual’s choice, to participate in the study or deny participation without losing their rights to health care. Participants were informed on their right to withdraw in the study at any time when they wish to do so. Informed consent was sought through signature or thumb print on consent forms. Due to inability of some study participants to read and write, informed consent was read clearly to participants by a research assistant, participants provided a verbal consent to participate in the study. Grade and provision of assent to participate in the study were the inclusion criteria for school children. Consent for children to participate in the study was sought from parents and guardians. The parents/guardians were invited by a local community health worker one day before the interview, the purpose of the study, procedures, harms and benefits were explained to parents and their children i.e. what study participation involved, including their right to withdraw without losing their rights to health services. Children who volunteered to participate in the study had to assent to be eligible. Only assenting children with parental or guardians’ consent were eligible to participate in the study. Participants were given a unique study identification number to ensure confidentiality of the data collected. All adult participants consented to participate into the study. Information obtained from the study was stored in a locked cabinet and a password protected digital database that only the research team was capable of accessing.

## Results

### Socio-demographic characteristics of the study participants

A total of 103 children, of whom 49.5% were female and 168 parents of whom 51.2% were female, participated in focus group discussions (FGDs). Of the children FGD participants, 48.5% belonged to Butini sub village, 26. 2% belonged to Bomani subvillage and 25.2% belonged to Miyu sub village. For parents, 26.8% belonged to Butini sub villages, 35.1% belonged to Bomani subvillage and 38.1% belonged to Miyu subvillage. Ten and 16 FGDs were conducted with children and parents, respectively (Table 1). Twenty nine parents and 20 children were involved in semi-structured interviews. Fifty percent (50%) of children participating in the semi-structured interviews were females while 14 (48.3%) of parent participants were females. All children were primary school pupils with age ranging from 12 to 15 years. Median age was 13 years. The parent’s age ranged from 25 to 80 years. Nineteen (66%) of the parents had primary school education, 7 (24.1%) were illiterate while only 3 (10%) had secondary school education. All participants belonged to the *Wasukuma* ethnic group and majority of the parents (93.1%) were subsistence farmers (Table 2).

**Table 1:** Distribution of focus group discussion participants by sex and sub-village

Sex	Children FGD (*n* = 10)			Parents FGD (*n* = 16)		
	Male No. (%)	Female No.(%)	Total No. (%)	Male No.(%)	Female No.(%)	Total No (%)
Sub village						
Miyu	13(12.6)	13(12.6)	26(25.2)	40 (23.8)	24(14.3)	64 (38.1)
Bomani	14(13.6)	13(12.6)	27(26.2)	21(12.5)	38(22.6)	59(35.1)
Butini	25(24.3)	25(24.3)	50(48.5)	21(12.5)	24(14.3)	45(26.8)
Total	52 (50.5)	51(49.5)	103(100)	82 (48.8)	86(51.2)	168 (100)

**Table 2:** Socio-demographic characteristics of interviewees in the semi structured interviews (SSI)

Variable	Children (*n* = 20) Frequency (%)	Parents (*n* = 29) Frequency (%)
Sex		
Males	10 (50)	15 (51.7)
Females	10 (50)	14 (48.3)
Age (yrs)		
12–13	15 (75)	
14–15	5 (25)	
≤ 25		5 (17.2)
25–34		11 (37.9)
35–44		4 (13.8)
45–54		5 (17.2)
≥ 55		4 (13.8)
Education		
Standard four	4 (20)	
Standard five	6 (30)	
Standard six	6 (30)	
Standard seven	4 (20)	
No formal		7 (24.1)
Primary		19 (65.5)
Secondary		3 (10.3)
Occupation		
Pupil	20 (100)	
Subsistence farmer		27 (93.1)
Other		2 (6.9)

#### Priority health problems in the community

Parents and children participants identified a number of diseases such as malaria, sexually transmitted infections including HIV/AIDS, gonorrhea and syphilis, fever, typhoid, sickle cell anemia, dehydration, pneumonia, coughs, headaches, tuberculosis, stomachaches, skin diseases, epidemics such as cholera, dysentery, and diarrhea, gut helminths, ulcers, pains (including joints, waist, back); swollen legs, eye and heart diseases as common health problems affecting the community. Very few participants mentioned schistosomiasis as a common health problem in the community.

The majority (78.5%) of parent respondents and 50% of children respondents revealed that schistosomiasis is a disease of no health priority in the community compared to other acute health problems. Disease priority at family level was given to diseases that are acute and life threatening such as malaria and cholera. Urinary schistosomiasis is given priority only when patients are seriously sick (Table 3). This was demonstrated by participants in interviews. One child reported: *“when we compare this with other diseases like malaria, the malaria patient must be treated first because the disease is more severe than urinary schistosomiasis. The person with urinary schistosomiasis will be treated when the condition is advanced”* [Children SSI]. Similarly a parent participant reported “*Our community does not grant schistosomiasis the priority it deserves, unlike cholera. There are those who go to hospitals but others visit traditional healers. Even myself I am suffering from urinary schistosomiasis but I am only using herbs to treat it. It is difficult to be open to others, therefore a patient treats it as a secret and sometimes even family members are not informed*.”[Parent SSI_male]. Another child added “*This disease is not given priority, other diseases like HIV/AIDS are given more priority and people strive to protect themselves against the disease, but with urinary schistosomiasis, the person suffering from it will be given priority only if she/he is passing blood in urine”* [Children SSI]. Likewise, one parent said, *“People do not take urinary schistosomiasis seriously because it is not an acute condition. It starts slowly and children between 5 and 16 years old get infected and healed after treatment. Some people think that the disease is hereditary and one can grow up with it. The disease affects mostly school children”* [Parents FGD_male]. Generally, adult participants were more likely to perceive urinary schistosomiasis as a disease of low priority compared to children.

**Table 3:** Comparison of responses between children and parents on semi structured interview

Variable	Children response (%) *s* = 20	Parents/guardians response (%) *n* = 29
Sex		
Female	10 (50%)	14 (48.3%)
Males	10 (50%)	15 (51.7%)
Age	12–15 years	25- 80 years
Schistosomiasis not perceived to be a major health problem	10 (50%)	22 (75.8%)
Transmission		
Completely wrong (hereditary, diet related)	13 (65%)	8 (27%)
Mixed up with gut helminth (eg bare foot around toilets)	14 (70%)	14 (48.3)
Correct that it involves water contact	15 (75%)	13 (44.8%)
Knowledge that snails transmit	8 (40)	9 (31%)
Perception that is man made through excrete disposal	16 (80%)	11 (37.9%)
Water contact through playing	19 (95%)	13 (44.8%)
Symptoms		
Abdominal pains	16 (80%)	19 (65.5%)
Passing of blood	16 (80%)	19 (65.5%)
Measures to prevent control		
Avoiding random excreta disposal in or near water water bodies (environmental hygiene)	6 (30%)	7 (24.1%)
Avoid playing/swimming in water	15 (75%)	9 (31%)
Snail control	1 (5%)	2 (6.8%)
Health education	5 (25%)	8 (27%)

#### Perceptions on transmission of urinary schistosomiasis

Discrepancies in schistosomiasis knowledge were revealed among both parents and children, with misconceptions on the real cause of schistosomiasis. Majority (65%) of child respondents and 27% of parent informants had incorrect knowledge on the transmission of urinary schistosomiasis. Informants associated urinary schistosomiasis with diet related infection by eating too much tamarind fruit, eating too much table salt, eating raw food, drinking dirty water or as a hereditary disease where children are born with schistosomiasis. Of the respondents interviewed, 70% of children and 48.3% of parent participants had no clear knowledge to distinguish between schistosomiasis and soil transmitted helminths. Participants pointed out that going to the toilet and bath rooms with bare feet as well as walking bare feet is a means of transmission of urinary schistosomiasis a perception which is incorrect (Table 3). One participant reported: *“Children in our community normally attend cattle. They walk long distances bare footed and therefore they are likely to get infected with urinary schistosomiasis”* [Children SSI _male]. On the same note, one participant said: “*We normally get urinary schistosomiasis because we walk bare feet, we don’t like walking like this but we don’t have shoes”* [children FGD females].

However, a few adult participants wrongly described urinary schistosomiasis as a sexually transmitted disease. As one parent reported: “Urinary *schistosomiasis is transmitted through having sex with the affected person and drinking dirty water*” [Parent SSI_male]. Other participants reported to know nothing about the transmission of the disease.

Of the total informants in the study, the majority of child participants (75%) did know that urinary schistosomiasis is transmitted through swimming in ponds and wells infested with schistosome cercariae.. Of the adult informants, only 44.8% reported to have the correct knowledge of urinary schistosomiasis transmission through water contact (Table 3).

In case of knowledge about transmission of the disease, only 40% of children participants reported that the disease is transmitted by snails. Of the parent informants, very few (31%) knew that snails present in water sources are responsible for the transmission of urinary schistosomiasis (Table 3). One standard six female pupil said: *“We children, normally play in ponds and wells, the water in these sources is not safe, it is full of micro organisms and snails that made us get urinary schistomiasis”.* In one focus group discussion, a participant mentioned: *“The presence of snails, children eat a lot of tamarind fruit, drink stagnant water and take baths in infested water pools.”*[Parents FGD_male].

Most (80%) of the child respondents perceived urinary schistosomiasis as a man made disease. Fewer (37.8%) adult respondents agreed that urinary schistosomiasis is a result of human behavior due to indiscriminate disposal of human waste products. Participants argued that had it not been for human insanitary behavior (indiscriminate excreta disposal) schistosome eggs would not have been introduced in water and the whole schistosomiasis life-cycle would not have begun (Table 3). One participant remarked: “*Indeed schistosomiasis is a man made disease because of non-use of latrines for defecating and urinating. Excreta disposal in or near water is responsible for the infection when people get into contact with infected water.”* [Parent SSI_female]. Children mentioned the issue of urinating in open spaces as the factor for the spread of urinary schistosomiasis from one person to another. One child reported: *“When an infected person urinates or defecates around or inside the water sources, this transmits urinary schistosomiasis because people use the same water for different uses like drinking, cooking, washing clothes and taking baths”* [Children FGD_female]*.* Another pupil said, *“If a person has urinary schistosomiasis and urinates in an open space and another person with bare feet step on it, he/she can get infected. If a urinary schistosomiasis infected person urinates in the water which is used by other people for drinking, taking a bath or for cooking, can infect others”* [Children FGD_male]. However, some participants disagreed with the disease being man made, one parent said, “*It is because of the environment we live in, as from time immemorial we have been using the same water from shallow wells and ponds. And it is not ‘man-made’ because snails are the cause.”* [Parents FGD_male].

#### Water contact practices

About three-quarters of the children participating in the study knew that water contact practices had impact on urinary schistosomiasis transmission, they highlighted that children have a habit of playing in water as part of recreational activities. In contrast, only 44.8% of parent informants reported knowing that swimming, playing and fishing in water ponds, is a risk behavior for transmission of urinary schistosomiasis among children (Table 3). The study participants reported that children prefer playing in water during the evenings. One pupil reported, *“Children play in the infested water during the evening because most of the time the rain comes in evening so we play in settled rain water, also in the evening we go to swim in the ponds as the owners of the ponds will have left already”* [Children FGD _female].

#### Symptoms of urinary schistosomiasis

Of the respondents interviewed, 65.5% of parents had correct knowledge of urinary schistosomiasis symptoms. They reported lower abdominal pain and passing blood in urine as the common symptoms for the disease. For children informants, the majority had adequate knowledge of the symptoms of urinary schistosomiasis (Table 3). However, symptoms like irritation and frequent urination were infrequently mentioned by respondents. Very few participants had no clear understanding of the disease symptoms. One adult participant said: “*Among the symptoms of urinary schistosomiasis include lower abdominal pains, lesions along urinary tract and then passing blood in urine and more often experiencing pain in the genitals, yellowish urine and painful urination”* [Parents FGD_male].

#### Measures to prevent and control urinary schistosomiasis

Study participants agreed that schistosomiasis is preventable. They mentioned a number of preventive measures of which 30% of school children and 24.1% of parent participants stated that avoiding random excreta disposal in or near water bodies, environmental hygiene and observing general cleanliness is an important strategy for overcoming transmission of urinary schistosomiasis. Seventy five percent of child participants and 31% of parents interviewed stated that avoiding playing in water and swimming in ponds and water pools will reduce transmission of urinary schistosomiasis. However, very few (5%) of child participants and 6.8% of parent participants mentioned snail control in the water sources through the use of chemicals (mollusciciding) as a means of reducing transmission (Table 3).

Over 27% of parents who participated in the study noted that health education among community members is the most important preventive measures against schistosomiasis. Twenty five percent of school children also reported that health education is necessary means of prevention against schistosomiasis (Table 3).

Participants went further by categorizing the measures to minimize contact with infested water in two ways, government and community efforts. For the government, participants suggested provision of deep protected wells and water taps as the most important and permanent alternative source of safe water in their local context. One child said “*The government has been trying to treat water sources in our communities but that is not enough, it has to be done frequently”* [Children FGD_female]. Another participant said *“The Government can even treat the same water that we are using, we are requesting the government to do that”.*

[Children FGD_male]. At community level, participants suggested construction and proper use of latrines and bathrooms as efforts to minimize contamination of water sources. It was suggested that for proper human excreta disposal, each household should construct and use latrines and bathrooms instead of using open spaces for urination.

To prevent urinary schistosomiasis, parents and children expressed a need for mutual efforts among community members. Most of the parents and children suggested that children should not be allowed to play, swim and fish in water bodies contaminated with human excreta, wearing protective shoes while working in water environments, boiling water and health education as measures to control urinary schistosomiasis among children. One child said: “*We are advising to have people who will act as watchmen in those ponds where children go to swim. This will scare children from going there to swim”* [Chldren FGD_male]. Furthermore, some of the study participants suggested the use of molluscicides for snail control so as to make their water sources safe. The majority of study participants agreed that urinary schistosomiasis is curable by modern medicines, by provision of praziquantel as an anti-schistosomal drug, whilst traditional sector of health care was considered to have insignificant role in the treatment of schistosomiasis. However, community health seeking behavior differs from one family to another and from one person to another. One parent participant remarked: *“If one suffers from schistosomiasis in most cases he/she is sent to the hospital for further treatment, or medicine is bought from essential drug shops* (*maduka ya dawa muhimu*) *and sometimes patients are sent to traditional healers”* [Parents FGD_male]. Some participants reported that they attended hospitals only when the disease was advanced. One child said *“A schistosomiasis patient is only taken to the hospital when his/her condition becomes severe and fails to walk alone, otherwise he/she will not be taken to the hospital”* [Children FGD_ male]. One of the children FGD participant reported, “*There is a traditional medicine which is known by local name as (Ntangala). Patients have to boil its roots mixing with water then drink”* [Children FGD_ female]. Another child participant added: *“It is true that schistosomiasis can be cured by traditional medicines. A patient has to drink two cups of the boiled medicine per day, one cup in the morning another in the evening for three days. My father is a traditional healer. He always treats people by using traditional medicines, he normally treats two to three patients per day and I witness people getting cured”* [Children SSI_Female].

## Discussion

For the successful and sustainable control of urinary schistosomiasis, the disease must be considered as a major public health problem in the community. To achieve this, community members needs to have the relevant knowledge, correct perceptions and to apply the correct preventive and control measures. This study was conducted with the intention to complement quantitative findings of a parasitological and malacological study conducted in Shinyanga district [25], by exploring individual and community social contexts of urinary schistosomiasis. The study used descriptive qualitative research inquiry design to solicit information from community members on knowledge, perceptions and practices of schistosomiasis transmission.

The study findings revealed that in Shinyanga, community members considered schistosomiasis as a disease of low priority. Participants reported acute diseases such as malaria and cholera as high priority diseases compared to schistosomiasis [35]. In Shinyanga district, this was attributed to the chronic nature of schistosomiasis where many years of infection often occurring before experiencing severe symptoms. A similar observation was reported by Kloos who argued that “*except for heavy infections, schistosomiasis is considered a relatively unimportant disease in many endemic areas. Affected communities tend to consider haematuria as a normal growth stage during childhood”* [36]*.* This suggests that both parents and children in the community were not well informed about the disease, consistent with findings from Kenya [37]. Control and eventual elimination of the disease could be very difficult if the affected community does not perceive the disease as a major public health problem [5, 6, 38, 39]. There is therefore a strong need to supplement parents and children’s knowledge on schistosomiasis to sufficiently raise disease awareness to a level that influences practices with an emphasis on behavioral change.

Most of the child respondents did, however, have correct knowledge on the transmission of urinary schistosomiasis, knowing that it occurs through water contact. Parents also had fair knowledge about true transmission modes of the disease. These findings were consistent with an observation from Ghana where over 60% of the community members knew that the disease was transmitted through water contact [40]. However, this knowledge was often combined with misconceptions whereby some respondents had misconceptions about the disease aetiology perceiving the disease to be hereditary, sexually transmited or attributing it to diet related infections. Similar misconceptions were reported by Mwanga et al in Magu district, Tanzania and by Bruun et et al. in the North-west part of the country [41, 42]. In Mozambique, 22% of participants reported that schistosomiasis is a sexually transmitted disease, while 12% thought that the disease is hereditary indicating that similar misconceptions can occur over wide geographical and cultural settings [43]. Amongst, child participants, misconceptions went further by associating the disease with eating too much tamarind fruit, too much table salt and drinking of dirty water. A similar observation where drinking of contaminated water was perceived to be among the cause of schistosomiasis infection has previously been reported [43, 44] Our study participants, particularly children also confused urinary schistosomiasis transmission with soil-transmitted helminth infections and reported that going to the toilet or bathrooms with bare feet as well as walking bare feet could lead to transmission of urinary schistosomiasis. However, overall, the majority of the study participants were more informed about the disease symptoms of urinary schistosomiasis. This observation is in line with other studies conducted elsewhere, where high knowledge of urinary schistosomiasis was reported [23, 40, 43, 45, 46].

Despite clear understanding amongst school children that water contact practices relate to transmission of urinary schistosomiasis, study respondents revealed that children were more exposed to schistosomiasis infection due to their active water contact to activities such as swimming, fishing and playing in infested water in line with findings of other studies [18, 35, 47–50].

Some study participants did suggest refraining from infested water as a preventive measure against schistosomiasis. However, without investment in water and sanitation infrastructure, this is not easy to achieve, as people have no other alternative sources of water to use [6]. It should be born in mind that improved access to safe water such as deep and protected wells; water taps and sufficient sanitation are effective in reducing schistosomiasis infection [51].

Parents and children health seeking behavior relied on both traditional and modern medicines. Based on the knowledge of disease symptoms and observable treatment outcome [52], modern treatment for urinary schistosomiasis was the preferred option over traditional medicine among most participants. This was influenced by increased effectiveness of modern medicines in curing the disease over herbal remedies. Similar observations were reported in Ghana and elsewhere in Tanzania [53, 54]. However, some participants opted for traditional medicine due to perceptions that the disease lacks serious impact and was assumed to be part of their normal life. Self-treatment was practiced by participants as they considered the disease to be a secret of an infected individual and shameful to speak about, even with other family members. This is consistent with findings reported in Ghana and elsewhere [43, 53, 55]. The use of combined therapies (traditional and modern) for schistosomiasis treatment was also reported in other studies [41, 56].

## Conclusions

A meaningful schistosomiasis control programme should take into account people’s knowledge, perceptions, attitudes and practices on the disease. Despite good knowledge, perceptions, attitudes and practices on schistosomiasis by a substantial segment of the study participants, this study revealed misconceptions and inadequate knowledge on the disease among participants in Shinyanga district which suggest that participants had limited information about the disease which in turn calls for health education campaigns as part of disease control interventions.. Collaborative efforts between the government and the community are required to reduce water contact behaviors that results in the transmission of urinary schistosomiasis. To make this feasible, health education and availability of alternative sources of clean and safe water are recommended to complement ongoing efforts to control schistosomiasis in Shinyanga district and other endemic areas.

## Additional files

Additional file 1: Focus group discussion (FGD). (DOCX 13 kb)

Additional file 2: Semi structured interviews (SSI). (DOCX 15 kb)

## Data Availability

The datasets used in this study are available from the corresponding author on reasonable request.

## Abbreviations

FGD: Focus group discussion
HBREC: University of Cambridge Human Biology Research Ethics Committee
HIV/AIDS: Human Immune Virus/Acquired Immune Deficiency Syndrome
NIMR: National Institute for Medical Research
SSI: Semi structured Interview
WASH: Water Sanitation and Hygiene
WHO: World health organization
