# Supplementary material for: Community knowledge, perceptions and water contact practices associated with transmission of urinary schistosomiasis in an endemic region: a qualitative cross-sectional study
Source: BMC Public Health. 2019 Jun 7;19:703. doi: 10.1186/s12889-019-7041-5 (PMC6554870; doi:10.1186/s12889-019-7041-5)
Supplement: Supplementary file 2 — Semi structured interviews (SSI). (DOCX 15 kb) [file 12889_2019_7041_MOESM2_ESM.docx]

**THE TRANSMISSION OF *SCHISTOSOMA HAEMATOBIUM* AMONG SCHOOL AGED CHILDREN IN SHINYANGA REGION, TANZANIA.**

***To be administered to parents/guardians and children***

**(Semi structured Interview (SSI) Schedule)**

**Form of Interview with participants (SSI)**

|  |
| --- |

1. Number of interviewee:

| DD/MM/YYYY |
| --- |

1. Date:

|  |
| --- |

1. Name of interviewer:

|  |
| --- |

1. Mode of recording:
2. **Individual details of interviewee**

| S/N | Question | Response |
| --- | --- | --- |
| 1 | Number of participant |  |
| 2 | Age (years only) |  |
| 3 | Tribe |  |
| 4 | Highest education |  |
| 5 | Occupation |  |
| 6 | Denomination |  |
| 7 | Ward |  |
| 8 | Village |  |
| 9 | Sub village |  |

1. ***GUIDELINE FOR INTERVIEW BETWEEN INTERVIEWER AND INTERVIEWEE***
2. What are the common health problem affecting people in the community? (Probe: which ones affect mostly children, how and why?)
3. Do you think that urinary schistosomiasis is a main concern health problem in this community? (Probe: why and what segments of population are mostly affected?)
4. What causes urinary schistosomiasis?(probe: interaction of human behavior, environment, snails, water contamination?).
5. What are the symptoms of urinary schistosomiasis?(Probe: urine with blood?)
6. How is urinary schistosomiasis transmitted?(probe: the role of human behavior and practice in schistosomiasis transmission)
7. Do you think in your community children have water contaminative behavior? **(Probe**: How and why children have water contaminative behavior?)
8. what should be done to reduce water contact in children?(probe:
9. Do you think that technical efforts to minimize water contacts by provision of alternative sources of safe water, safe laundry sites, safe public baths, latrines and bridges will help to change people’s contaminative behavior? (**Probe:** the role of traditional water contact in this regard?)
10. Do you think that behavioral change, health education efforts to enhance community participation and compliance with the national schistosomiasis control programme will ensure successful and persistent control of the infection?
11. It is believed that schistosomiasis is a man made disease in the sense that its causation and transmission is encouraged by man’s unsanitary behavior. Do you agree/ disagree with this assertion?(Give reasons)
